# High Water Density at Non-Ice-Binding Surfaces Contributes to the Hyperactivity of Antifreeze Proteins

**DOI:** 10.1021/acs.jpclett.1c01855

**Published:** 2021-09-07

**Authors:** Akash Deep Biswas, Vincenzo Barone, Isabella Daidone

**Affiliations:** † Scuola Normale Superiore di Pisa, Piazza dei Cavalieri 7, Pisa 56126, Italy; ‡ Department of Physical and Chemical Sciences, University of L’Aquila, via Vetoio (Coppito 1), 67010 L’Aquila, Italy; ¶ National Institute for Nuclear Physics (INFN) Pisa Section, Largo Bruno Pontecorvo 3, 56127 Pisa, Italy

## Abstract

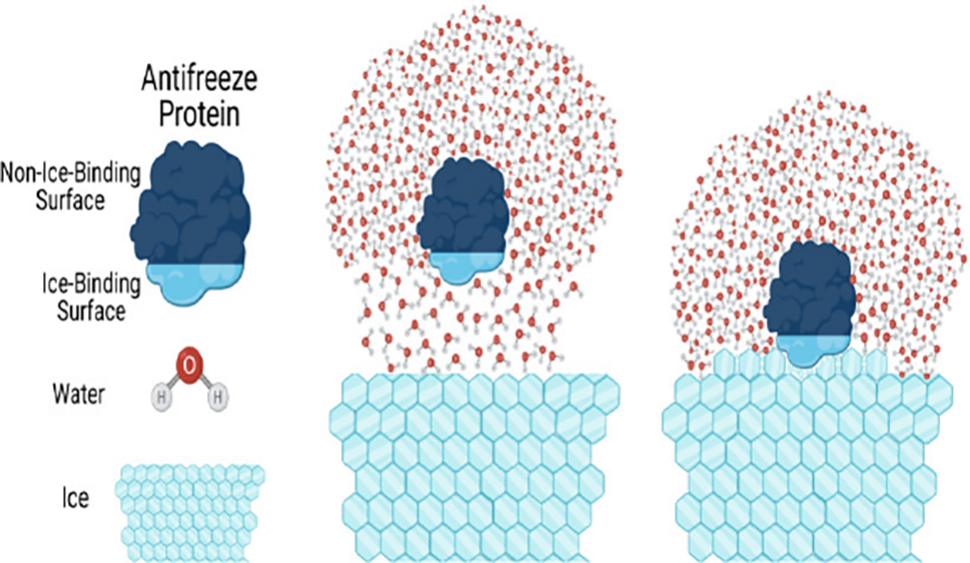

Antifreeze proteins (AFPs) can bind to ice nuclei thereby inhibiting their growth and their hydration shell is believed to play a fundamental role. Here, we use molecular dynamics simulations to characterize the hydration shell of four moderately-active and four hyperactive AFPs. The local water density around the ice-binding-surface (IBS) is found to be lower than that around the non-ice-binding surface (NIBS) and this difference correlates with the higher hydrophobicity of the former. While the water-density increase (with respect to bulk) around the IBS is similar between moderately-active and hyperactive AFPs, it differs around the NIBS, being higher for the hyperactive AFPs. We hypothesize that while the lower water density at the IBS can pave the way to protein binding to ice nuclei, irrespective of the antifreeze activity, the higher density at the NIBS of the hyperactive AFPs contribute to their enhanced ability in inhibiting ice growth around the bound AFPs.

Surviving in exceptionally cold environments where temperatures drop below the melting temperature of ice is an example of an exclusive adaptive mechanism which protects the cells of a variety of organisms, including fish, insects, bacteria, plants, and fungi, from getting damaged at temperatures below zero. These organisms exploit a group of proteins, called antifreeze proteins (AFPs), that are able to lower the freezing temperature of the surrounding water. Despite their variability in terms of primary, secondary, and tertiary structures, AFPs share a common mechanism of action which consists of binding to small ice nuclei, preventing their further growth at the adsorbed positions.^1,2^

Binding of AFPs to specific ice planes is ruled by both hydrogen bonds and hydrophobic interactions with a complementary protein surface, termed the ice-binding surface (IBS). The mechanism proposed for the antifreeze activity is that, by binding to a growing ice nucleus, AFPs cause a microcurvature of the ice surface between the adsorbed AFPs.^1,3^ Because the ice-crystal growth is thermodynamically less favorable on a curved ice surface than on a flat one, the ice growth stops until the temperature drops. This leads to a local difference between the freezing and melting temperature, *ΔT*, known as thermal hysteresis (TH). On the basis of the TH values, the AFPs can be classified into moderately active (*ΔT* < 1 K), mostly alanine-rich α-helical AFPs, and hyperactive AFPs (*ΔT* > 1 K), mostly threonine-rich β-helical proteins found in insects. At low concentrations (<0.5 g/L), hyperactive AFPs greatly outperform more traditional antifreeze agents, making them of potential interest for use in medicine, agriculture, food processing, and surface protection.^4^

A general consensus has been recently reached on the nature of the interactions that determine the binding to the ice surface: a spatial match between the polar residues of the IBS and the oxygen–oxygen distance in the ice lattice is needed,^5−9^ pointing to a critical role for hydrogen bonds. At the same time, a relevant hydrophobic content in the IBS is required, providing an entropically driven thermodynamic contribution to ice binding. Although a crucial role played by the AFP’s hydration layer in the molecular mechanism of the antifreeze activity is recognized,^10,11^ its structural and dynamical characterization is still incomplete. On the basis of molecular dynamics (MD) simulations, it has been proposed that hydration water at the IBS could form an ice-like layer already in solution, thereby easing ice recognition.^12−19^ However, other MD simulations^20−23^ and an NMR study^24^ did not find any indication of ice-like structure in liquid water around AFPs. Concerning the dynamics, there is both computational^15,25^ and experimental evidence^11^ that water near the IBS displays exceptionally slower hydrogen bond reorientation dynamics compared with other protein surfaces.

At variance with the IBS, detailed studies of the role of the non-ice-binding surfaces (NIBSs) of AFPs are few. Previous computational work^18,22,26−28^ showed that the solvent at the IBSs features a lower density increment with respect to the bulk density and a higher degree of order with respect to the solvent at the NIBSs. In particular, we recently showed for two AFPs that although the overall hydration shell density does not differ from the one of non-AFPs,^22,23^ the higher content of hydrophobic (at IBS) and hydrophilic (at NIBS) residues in AFPs determines a slightly inhomogeneous density inside the hydration shell of AFPs (lower at IBS and higher at NIBS).^22^ While it is widely accepted that the lower density at the IBS favors binding to ice, the higher water density at the NIBS might discourage ice growth around the bound AFPs, as was suggested many years ago but on the basis of dynamical analysis,^15^ thus contributing to the curvature of the ice surface at the basis of TH. The coupled features of ice-binding and non-ice-binding surfaces in AFPs and the resulting density unbalance could thus contribute to the antifreeze activity by providing protection against ice growth.

Here, we provide further evidence of a clear correlation between the hydration density at the IBS and the antifreeze activity for four moderately active samples. More importantly we show that, rather than differences in the hydration structure at the IBSs, what actually discriminates between moderately active and hyperactive AFPs is an increased hydration density at the NIBSs of the latter. This increased density around the non-ice-binding parts of the hyperactive AFPs might contribute to enhance their capability of preventing the adsorbed protein from being overgrown by a growing ice surface.

Eight AFPs (four moderately active and four hyperactive) are simulated at a constant pressure and at room temperature (see Methods). The moderately active AFPs are the snow mold fungus AFP from *Typhula ishikariensis* isoform 6 (*Tis*AFP6, PDB ID: 3VN3), the fish type I AFP from *Pseudopleuronectes americanus* (*Pa*AFP, PDB ID: 1WFA), the fish type III AFP from *Zoarces americanus* (*Za*AFP, PDB ID: 1HG7) and the snow mold fungus AFP from *Typhula ishikariensis* isoform 8 (*Tis*AFP8, PDB ID: 5B5H). The hyperactive AFPs are the spruce budworm AFP from *Choristoneura fumiferana* isoform 337 (*Cf*AFP337, PDB ID: 1L0S), the beetle AFP from *Tenebrio molitor* (*Tm*AFP, PDB ID: 1EZG), the longhorn beetle AFP from *Rhagium inquisitor* (*Ri*AFP, PDB ID: 4DT5), the spruce budworm AFP from *Choristoneura fumiferana* isoform 501 (*Cf*AFP501, PDB ID: 1M8N).

In order to characterize their hydration shell, the proteins are modeled as ellipsoids (details are given in the Supporting Information), and the solvent density, ρ, around them is calculated as a function of the distance from the ellipsoid surface (see Figures S1 and S2 in the Supporting Information). To compare the properties of water within the hydration shell with those of the bulk water, a previously employed strategy^22,23^ was followed, which is based on a fictitious hydration shell filled with water molecules distributed as they would be in the bulk solvent. This strategy permits removal of possible effects arising from the different topological features of the protein surfaces on the hydration shell features and hence the removal of the effect of the spatial arrangement of the protein atoms that protrude from the protein ellipsoid surface on the density profiles. More details on the computational procedure are presented in Methods.

The variations of the hydration density with respect to bulk water are followed by computing the ratio ρ/ρ_b,fict_, in which ρ_b,fict_ is the density within the fictitious hydration shell as a function of the distance from the protein ellipsoid surface (see Figure 1, panel A). It is apparent that the solvent density at the protein surface is higher than the bulk density and reaches this latter limiting value (∼33.3 molecules nm^–3^ at 300 K) at around 1 nm from the protein surface. Furthermore, our results confirm that, as previously reported for several non-AFPs and a few AFPs, the ratio ρ/ρ_b,fict_ is always greater than 1; that is, the solvent density around the proteins is always higher than in the bulk.^22,23^

**Figure 1:**
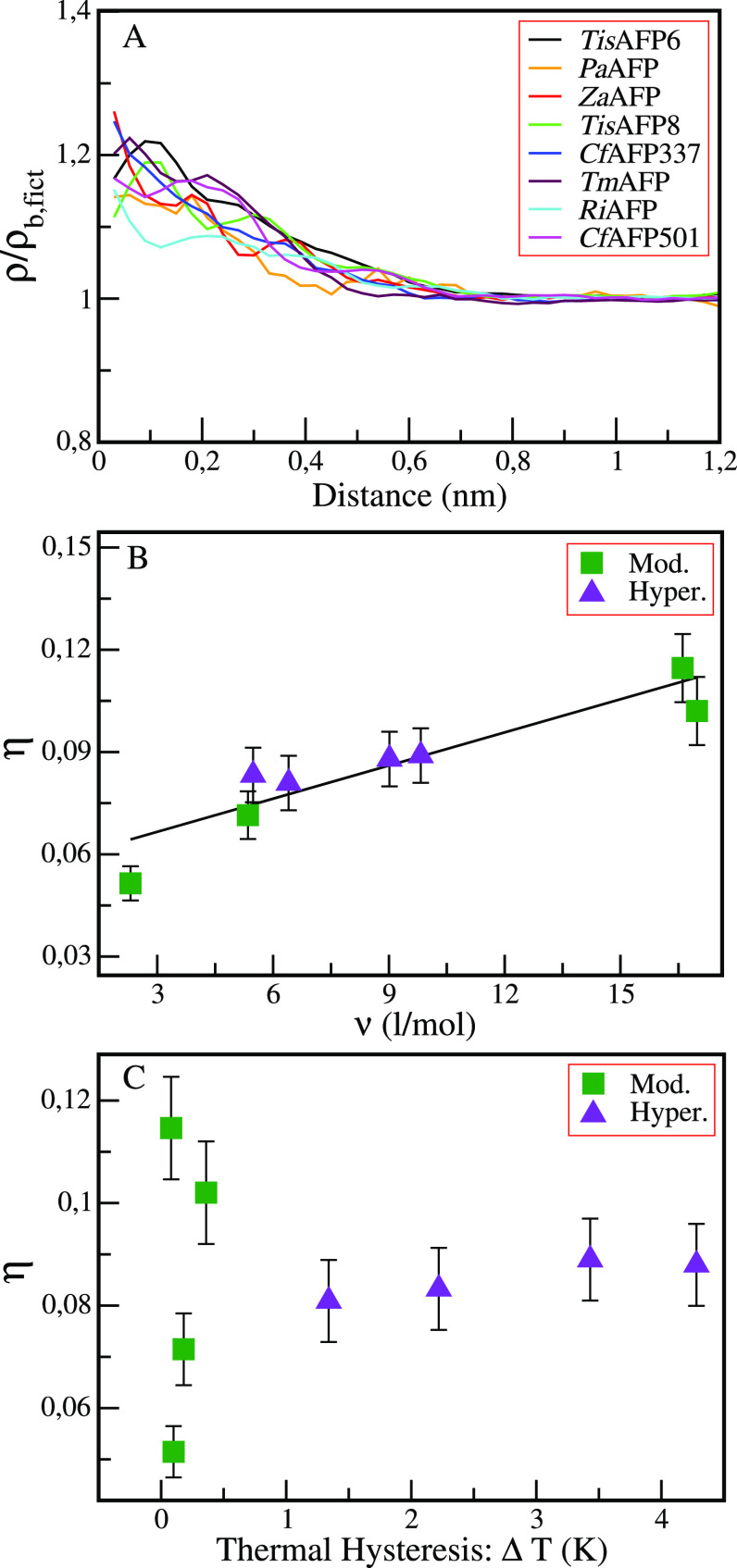
(A) Ratio ρ/ρ_b,fict_ as a function of the distance from the protein ellipsoid surface calculated for the eight proteins: *Tis*AFP6 (black), *Pa*AFP (orange), *Za*AFP (red), *Tis*AFP8 (green), *Cf*AFP337 (blue), *Tm*AFP (maroon), *Ri*AFP (cyan), and *Cf*AFP501 (magenta). (B) Relative surface density increment η as a function of partial molar volume *v* calculated for each protein molecule using eq 3. (C) η as a function of the antifreeze activity, *ΔT* (*K*), measured at a protein solution concentration of 0.3 g/L.^29^ The moderately active (Mod.) AFPs are represented with green squares, whereas the hyperactive (Hyper.) AFPs are represented with purple triangles.

In order to define the protein hydration shell thickness, and corresponding shell volume *V*_shell_, we use the distance from the protein ellipsoid surface of 1 nm at which the layer solvent-density reaches the bulk plateau value. The relative density increment, η, inside the hydration shell with respect to the bulk density, ρ_bulk_, is calculated as
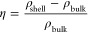
1where ρ_shell_, the density within the hydration shell, is estimated as
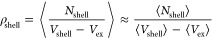
2In the last equation, *V*_ex_ is the protein-excluded volume (i.e., the volume enclosed by the solvent-accessible surface obtained by using a probe radius of 0.14 nm, according to the method reported in Eisenhaber et al.^30^); *N*_shell_ is the number of water molecules inside the hydration shell, and angle brackets indicate ensemble averages. It is noteworthy that exact and approximated formulations lead to identical results within the estimated error bar (the ρ_shell_ of all the studied proteins is reported in Table S1 of the Supporting Information). From the above data, we can also estimate the partial molar volume, *v*, through the following expression, which was previously shown to be affected by small statistical errors^31^ (its derivation is given in the Supporting Information):
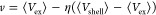
3

The values of the relative increment in the hydration-shell density, η, and of the partial molar volumes, *v*, along with the quantities needed for their calculation, for the eight studied AFPs are reported in Table 1. η is in the range of 6–12%, which is in agreement with experimental data^39^ and previous calculations.^22,23,40,41^ When η is reported as a function of the partial molar volume *v* (see eq 3), a clear correlation can be seen, indicating a length scale dependence of the density increase on the protein size (see Figure 1B). The dependence of η on the protein size was addressed in detail in a previous paper on a larger set of 18 proteins, out of which 8 were AFPs and 10 non-AFPs.^23^ Conversely, no clear correlation is observed between η and the AFP activities (see Figure 1 in which η is reported as a function of the antifreeze activity, i.e., the experimental thermal hysteresis *ΔT* (*K*) at a protein solution concentration of 0.3 g/L).

**Table 1:** Total Number of Residues (RN), Experimental Thermal Hysteresis (*ΔT*), Mean Excluded Volume (⟨*V*_ex_⟩), Mean Hydration Shell Volume (⟨*V*_shell_⟩), Number of SPC Molecules Inside the Hydration Shell (⟨*N*_shell_⟩), Hydration-Shell Density Increment Relative to Bulk Density (η), and Partial Molar Volume (*v*) as Obtained from the MD Trajectories of the Eight Proteins^a^

protein	RN	*ΔT*	⟨*V*_ex_⟩	⟨*V*_shell_⟩	⟨*N*_shell_⟩	η	*v*
*Tis*AFP6	222	0.08	34.57	95.58	2266	0.114	16.61
*Pa*AFP	37	0.10	5.78	43.71	1329	0.051	2.30
*Za*AFP^b^	66	0.18	11.50	48.07	1305	0.071	5.35
*Tis*AFP8	223	0.36	34.46	95.85	2254	0.102	16.98
*Cf*AFP337	87	1.34	13.81	53.16	1417	0.081	6.40
*Tm*AFP	82	2.22	12.24	49.87	1358	0.082	5.48
*Ri*AFP	139	3.43	21.16	75.82	1983	0.089	9.82
*Cf*AFP501	120	4.28	19.11	66.18	1707	0.088	9.01

a The hydration shell thickness is taken as 1 nm. *V*_ex_ and *V*_shell_ are reported in nm^3^. The relative density increment, η, with respect to the bulk density and the partial molar volumes, *v* (reported in l/mol), are calculated according to the eqs 1 and 3, respectively. The error on ⟨*V*_ex_⟩ and ⟨*V*_shell_⟩ is ∼0.3%, on ⟨*N*_shell_⟩ ≈ 0.2%, and the one on η is ∼3–4%. Errors on these quantities are calculated through the standard error of their mean evaluated over 3 subtrajectories.

b The number of water molecules inside the hydration shell of ZaAFP (i.e., 1305) is in good agreement with the experimental estimate of a lower limit of 1100 molecules.^38^

The lack of any significant correlation between the whole hydration-density of the AFPs and their activity prompted us to analyze the local density around the IBS, which is the specific surface involved in ice binding, and compare it to that around the NIBS, i.e., the rest of the protein surface (see Figure 2 for the definition of the IBS and the NIBS). In order to remove possible effects arising from the topological properties of the IBSs, the eight AFPs studied here were chosen to have rather flat IBSs with a similar surface size (see Table 2 and Figure S3 in the Supporting Information).

**Figure 2:**
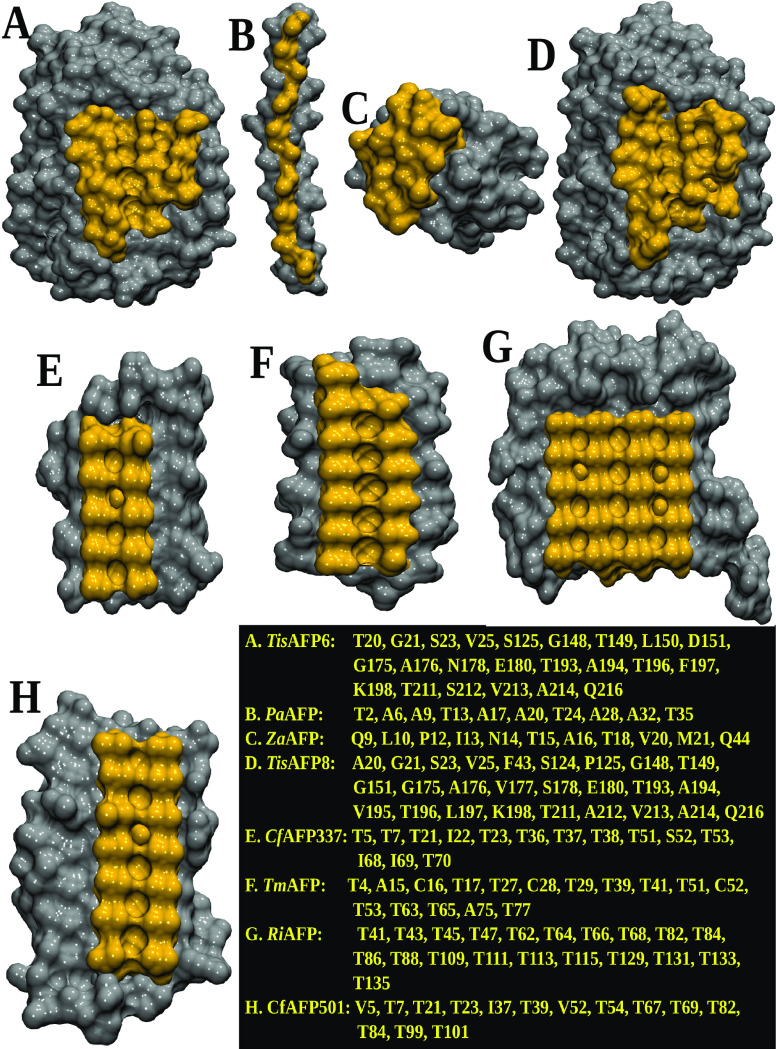
Ice-binding surface of each protein is colored in orange, and the rest of the protein surface, considered as NIBS, is colored in silver. The data contained in the original papers giving the crystal structures were employed to define the residues belonging to the IBS (reported in the inset): (A) *Tis*AFP6,^32^ (B) *Pa*AFP,^33^ (C) *Za*AFP,^34^ (D) *Tis*AFP8,^35^ (E) *Cf*AFP337,^7^ (F) *Tm*AFP,^6^ (G) *Ri*AFP,^36^ and (H) *Cf*AFP501.^37^

**Table 2:** Average Solvent-Accessible Surface Area, *S*, of the Whole Protein (WP) and of the Ice-Binding Surface (IBS) and Average Hydrophobic-Surface Fraction, *S*_pho_/*S*, for the WP, IBS, and the Non-Ice-Binding Surface (NIBS)^a^

	*S*		*S*_pho_/*S*		
protein	WP	IBS	WP	IBS	NIBS
*Tis*AFP6	99.87	10.11	0.56	0.56	0.56
*Pa*AFP	30.31	7.60	0.54	0.64	0.51
*Za*AFP	40.09	8.17	0.58	0.67	0.56
*Tis*AFP8	98.25	10.70	0.59	0.70	0.57
*Cf*AFP337	48.56	8.02	0.52	0.58	0.51
*Tm*AFP	42.68	7.57	0.46	0.61	0.42
*Ri*AFP	72.97	15.46	0.52	0.54	0.52
*Cf*AFP501	62.85	8.66	0.49	0.67	0.47

a The units of *S* are nm^2^. The *S* and *S*_pho_/*S* values are averaged over 10 000 structures extracted from the 100 ns MD trajectory of each protein. The error on the *S* values is ∼0.2% and on the *S*_pho_/*S* values is ∼4%.

For each AFP, the solvent-density around a given surface, ρ_surf_, is defined as the number of water molecules, *N*_w_, within a 0.55 nm radius cutoff from the heavy atoms on the selected surface per unit of SASA (ρ_surf_ = ⟨*N*_w_⟩/⟨*S*⟩ with *N*_w_ and the SASA, *S*, averaged along the MD trajectory). We chose a 0.55 nm cutoff, which is less than the 1 nm thickness of the whole hydration shell, to define the first hydration layer with the aim of maximizing the local differences in water density at the different surfaces (see Figure S4 in the Supporting Information for more details). The surface density calculation is also performed for the “fictitious” configurations in which the first hydration layer is filled with bulk water (ρ_surf_^fict^). In this way the local surface density of the IBS and NIBS can be compared among the different proteins by calculating the relative increment with respect to the bulk, η_surf_, as
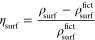
4η_surf_ is calculated for both the IBS and the NIBS of each protein, and the results are reported in Table 2 and Figure 3. For all the surfaces considered, a local increment with respect to bulk density, in line with the results on the whole hydration shell reported in Figure 1, is observed, suggesting that no ice-like structure (i.e., with a density lower than that of bulk) is present in the vicinity of the ice-binding surface. Moreover, the IBSs feature on average a higher η_surf_ with respect to the NIBSs, and for each protein, the IBS always displays a lower relative increment with respect to the corresponding NIBS (with the only exception being *Tis*AFP6 for which they are comparable).

**Figure 3:**
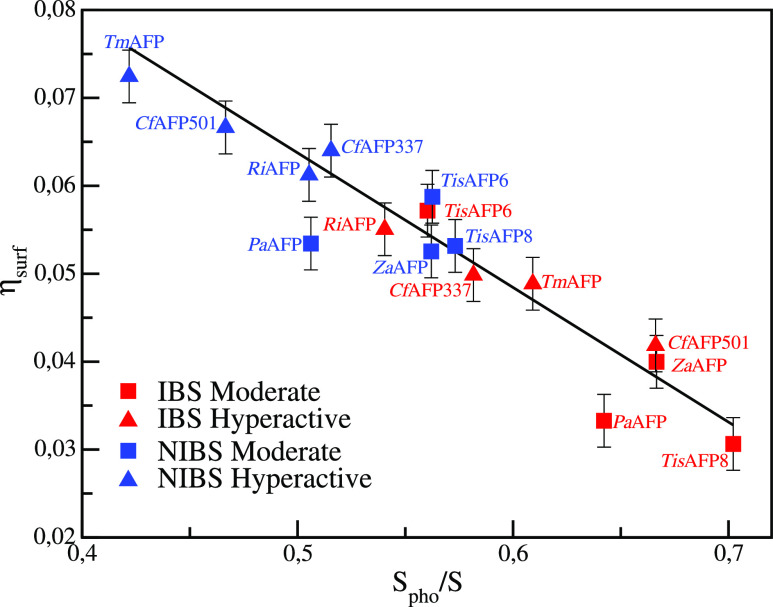
Change in the relative water-density increment with respect to the bulk (η_surf_) as a function of the hydrophobic fraction of the solvent-exposed-area (*S*_pho_/*S*) of the IBSs (represented in red) and NIBSs (represented in blue) of the eight antifreeze proteins.

Further analysis shows that the surface density increment with respect to the bulk density correlates with the chemical nature of the exposed surface (Table 3). From Figure 3, in which η_surf_ is reported as a function of the fraction of the accessible hydrophobic surface area, *S*_pho_/*S*, for all the IBSs and NIBSs, it can be seen that η_surf_ is inversely proportional to *S*_pho_/*S*. A lower increment corresponds to a higher *S*_pho_/*S*, and the ice-binding surfaces display the highest hydrophobic content. Moreover, a highly hydrophobic IBS is coupled to a highly hydrophilic NIBS, conferring to the AFPs a strong amphipathic character.^42^ Similar results were previously found on a smaller set, namely two of the eight AFPs studied here.^22^

**Table 3:** Relative Surface Density Increment η_surf_ of the IBS and NIBS^a^

	η_surf_	
protein	IBS	NIBS
*Tis*AFP6	0.056	0.057
*Pa*AFP	0.033	0.053
*Za*AFP	0.038	0.055
*Tis*AFP8	0.032	0.054
*Cf*AFP337	0.052	0.064
*Tm*AFP	0.047	0.077
*Ri*AFP	0.059	0.063
*Cf*AFP501	0.040	0.069

a The error on the η_surf_ is ∼3–4%.

In order to understand whether the properties of the local densities and the chemical character of the IBSs and NIBSs can be related to the antifreeze activity of the AFPs, we report η_surf_ and *S*_pho_/*S* as a function of *ΔT* (see Figure 4). For both moderately active and hyperactive AFPs, the hydrophobicity of the IBS is not only high (with *S*_pho_/*S* in the range of 0.54–0.70) but also similar between the two classes (being on average slightly higher for the moderately active ones). On the other hand, the hydrophobic character of the NIBS (lower than that of the corresponding IBS) is significantly higher for the moderately active than for the hyperactive AFPs.

**Figure 4:**
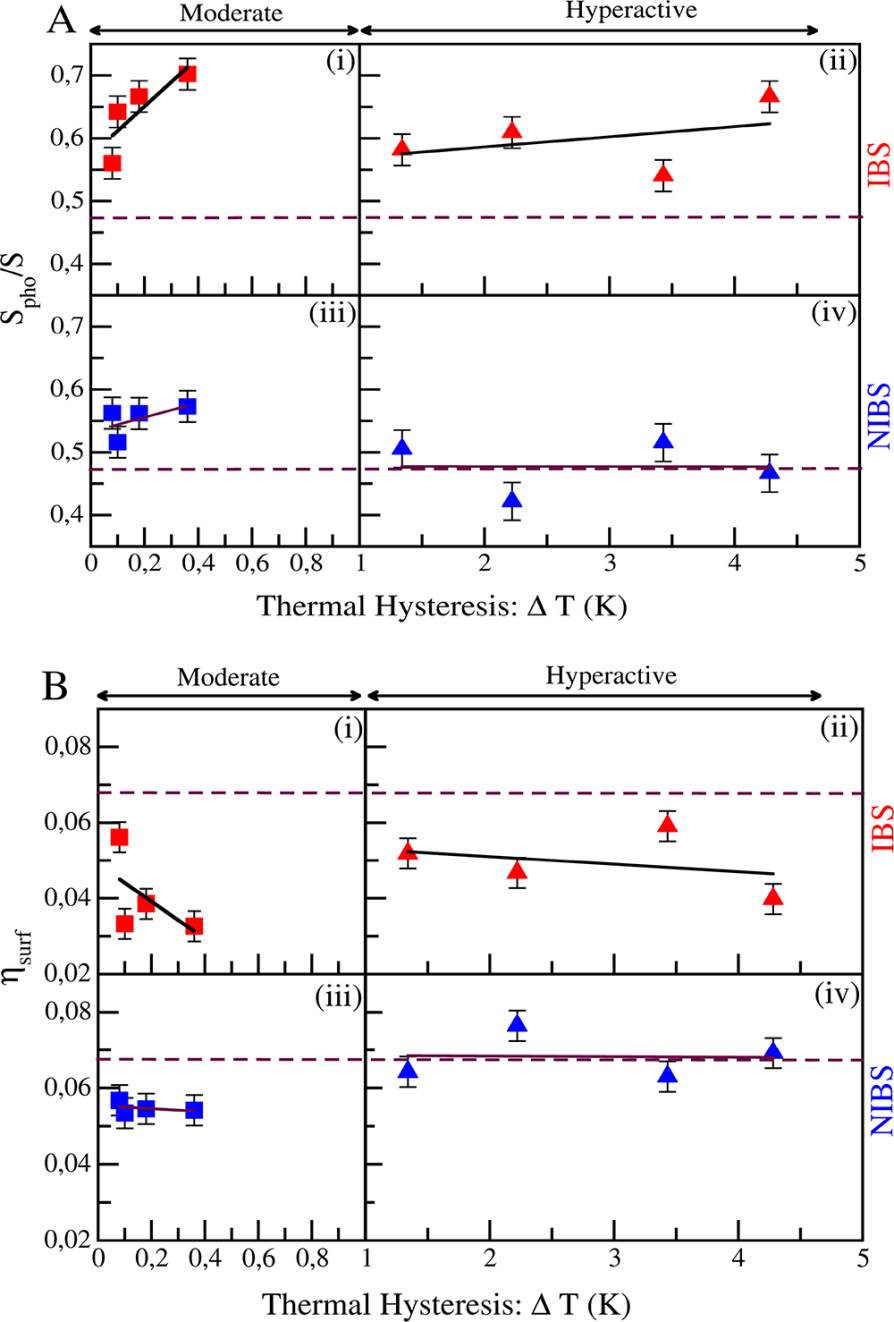
(A) *S*_pho_/*S* and (B) η_surf_ of IBS (red) and NIBS (blue) reported as a function of the antifreeze activity of the corresponding protein. The horizontal dashed lines in panels A and B indicate the average *S*_pho_/*S* and average η_surf_, respectively, calculated over the NIBSs of the four hyperactive AFPs.

Together with the strong correlation between η_surf_ and *S*_pho_/*S* shown above, similar results are obtained also for the relative increment of solvent density: while around the IBSs the η_surf_ is similar between moderately active and hyperactive AFPs, with on average slightly lower values for the moderately active AFP, around the NIBS η_surf_ differs, being higher for the hyperactive AFPs.

These results show that what actually differentiates moderately active from hyperactive AFPs are the properties of the NIBS, rather than those of the IBS. Indeed, for both classes of proteins the IBS has a high hydrophobic content, and a consequent lower water density, as previously reported by different groups.^15,22,26,43,44^ However, here we show that rather than this, it is the higher hydrophilic content and consequent higher relative density increase of the NIBS that differentiate the hyperactive AFPs from the moderately active ones.

## Methods

The MD simulations of the eight proteins were performed with the help of the Gromacs 5.1.4 software^45^ in conjunction with the OPLS-AA^46^ and SPC^47^ force fields for the proteins and water, respectively. The neutrality of all the simulated systems was ensured by adding (when needed) the suitable number of counterions. A constant temperature of 300 K was always enforced by the velocity-rescaling algorithm.^48^ An isothermal–isochoric (*NVT*) reference simulation of 100 ns was first performed for pure SPC water at 300 K with a density corresponding to the experimental liquid water density at the same temperature (∼33.3 molecules per nm^3^). The resulting pressure of ∼560 bar was then used to calibrate the densities of the boxes employed to simulate (in the *NVT* ensemble) the different proteins in SPC water. All bond lengths were frozen by means of the LINCS algorithm,^49^ thus allowing the safe use of an integration step of 2 fs. The particle mesh Ewald method^50^ was used to compute long-range interactions above a cutoff radius of 1.1 nm. After equilibration of the isolated protein and then of the full solute–solvent system, production simulations of 100 ns were run for all the systems.

The MD simulation of the pure SPC water described above was used to create the fictitious protein + bulk-water configurations. For each AFP, the protein coordinates extracted from 10 000 frames of the protein–solvent MD simulation were overlaid on 10 000 configurations extracted from the bulk-water simulation, and the water molecules overlapping the protein (i.e., with any distance between water and protein atoms lower than the sum of the corresponding van der Waals radii) were removed. This procedure provides 10 000 “fictitious” configurations of protein–solvent in which the hydration shell was filled with bulk-like water molecules. The solvent density, ρ_b,fict_, as a function of the distance from the protein ellipsoidal surface was then calculated for these fictitious “protein + bulk-solvent” configurations with the same procedure employed for conventional simulations. Details about the calculation of the protein hydration shell densities are provided in the Supporting Information (Section: Protein hydration shell density calculation).

The possible role of the specific force field employed in the MD simulations in determining the calculated densities was assessed analyzing two previous simulations for the moderately active *Za*AFP and the hyperactive *Cf*AFP501 proteins performed with the TIP4P/2005 model for water in conjunction with the amber03 force field for the protein.^51^ The results collected in Table S2 of the Supporting Information show that the relative density-increment in the hydration shell with respect to the bulk density, η, obtained by the above force field and by the SPC/OPLS-AA model employed in the present Letter are comparable concerning both the absolute η values for each protein and of the difference of η between the two proteins (0.017 and 0.016, respectively).

The fraction of the hydrophobic SASA was calculated with the GROMACS built-in module *gmx sasa*, which employs a definition of polar (hydrophilic) and nonpolar (hydrophobic) surface areas based on atomic partial charges (atoms with partial charges between −0.2 and 0.2 are considered nonpolar). A test of the dependence of the fraction of the hydrophobic SASA on the choice of the hydrophobicity definition is reported in Figure S5 of the Supporting Information showing that differences arising from this choice are small and systematic, thus not influencing appreciably the comparison between different proteins.

## Supporting Information Available

Details on the calculation of the protein hydration shell density and the partial molar volume; figures showing the protein structures and the corresponding hydration layers, hydration-layer density profiles, SASAs of the IBSs, radial distribution functions of water molecules around the aliphatic carbon atoms at the surface of the proteins, and fractions of hydrophobics SASA computed by means of two different definitions of hydrophobicity; tables reporting the average solvent density within the hydration shells of the proteins and showing a comparison between the density increase with respect to bulk water obtained from MD simulations of two AFPs with two different force fields (PDF)
