# Supplementary material for: High Water Density at Non-Ice-Binding Surfaces Contributes to the Hyperactivity of Antifreeze Proteins
Source: J Phys Chem Lett. 2021 Sep 7;12(36):8777–83. doi: 10.1021/acs.jpclett.1c01855 (PMC8450935; doi:10.1021/acs.jpclett.1c01855)
Supplement: Supplementary file 1 — jz1c01855_si_001.pdf [file jz1c01855_si_001.pdf]

High Water Density at Non-Ice-Binding Surfaces Contributes to the Hyperactivity of Antifreeze Proteins

Akash Deep Biswas,[†,‡] Vincenzo Barone,[†,¶] and Isabella Daidone[*,‡]

†*Scuola Normale Superiore di Pisa, Piazza dei Cavalieri 7, Pisa, 56126, Italy*

‡*Department of Physical and Chemical Sciences, University of L'Aquila, via Vetoio (Coppito 1), 67010 L'Aquila, Italy*

¶*National Institute for Nuclear Physics (INFN) Pisa Section, Largo Bruno Pontecorvo 3, 56127, Pisa, Italy*

E-mail: isabella.daidone@univaq.it

Phone: +39-338-654-5853

Supporting Information

A Protein Hydration Shell Density Calculation

The next paragraph summarizes the procedure used to calculate the solvent density profile around the protein within layers at increasing distances from the surface of the ellipsoid used to model the protein molecule both along a standard molecular dynamics (MD) simulation and for the ensemble of fictitious "protein + bulk-solvent" configurations generated as described in the main text of the manuscript. In order to characterize the solvent density around the protein, the latter is modeled by an ellipsoid, the axes of which are defined, for

each MD time frame, by the eigenvectors and eigenvalues of the 3×3 geometrical covariance matrix of the x, y, z atomic coordinates.[1] A set of ellipsoidal layers around the protein, defined by the consecutive ellipsoids with semi-axes $a_i^{(n)} = a_i + n\delta$ with fixed increment $\delta = 0.03$, is then considered. The solvent density at increasing distance from the protein ellipsoid is obtained by calculating at each MD frame the instantaneous water density within each of these layers (disregarding the possible presence of protein atoms and/or counterions) and averaging over the MD trajectory (the corresponding solvent-density profiles are reported in Figure S2 for all studied proteins). The distance from the protein surface at which the solvent density reaches the bulk value ($\approx$ 33.3 molecules per nm^3 at 300 K) defines the hydration shell boundary layer, i.e., the thickness of the protein hydration shell and corresponding shell volume V_{shell}.

Following the procedure suggested by Eisenhaber et al.,[2] the volume V_{ex} enclosed by the solvent-accessible surface (the so-called protein excluded volume), can be obtained by means of the Gromacs package using a probe radius of 0.14 nm. The mean protein volume and the corresponding thermal distribution are then obtained by averaging the V_{ex} values computed at each MD time frame. From V_{shell}, V_{ex} and the number of SPC water molecules inside the hydration shell, N_{shell}, the mean solvent density within the accessible volume of the protein hydration shell can be obtained according to:

$$\rho_{shell} = \left\langle \frac{N_{shell}}{V_{shell} - V_{ex}} \right\rangle \approx \frac{\langle N_{shell} \rangle}{\langle V_{shell} \rangle - \langle V_{ex} \rangle} \tag{1}$$

Comparison of the mean solvent density inside the hydration shell with the bulk solvent density ρ_{bulk}, provides the corresponding relative density increment η:

$$\eta = \frac{\rho_{shell} - \rho_{bulk}}{\rho_{bulk}} \tag{2}$$

B Partial Molar Volume Calculation

The protein partial molar volume, i.e. $v = (\partial V/\partial N)_{p,T,N_{solvent}}$ with V the volume of the system, N the number of solute molecules, at a constant pressure p, constant T, was calculated as:[3]

$$v = \langle V_{ex} \rangle + \frac{\langle N_{shell} \rangle}{\rho_{shell}} + \frac{(N_{SPC} - \langle N_{shell} \rangle)}{\rho_{bulk}} - \frac{N_{SPC}}{\rho_{bulk}} \tag{3}$$

where N_{SPC} denotes the total number of SPC water molecules contained within each protein simulation box. Equation 3 can be arranged as follows:

$$\begin{aligned} v &= \langle V_{ex} \rangle + \langle N_{shell} \rangle \left(\frac{1}{\rho_{shell}} - \frac{1}{\rho_{bulk}} \right) \\ &= \langle V_{ex} \rangle + \frac{(\rho_{bulk} - \rho_{shell})}{\rho_{bulk}} \frac{\langle N_{shell} \rangle}{\rho_{shell}} \\ &= \langle V_{ex} \rangle - \eta(\langle V_{shell} \rangle - \langle V_{ex} \rangle) \end{aligned} \tag{4}$$

where η is the relative density increment inside the hydration shell with respect to the bulk density described in the previous section.

C Supplementary Figures

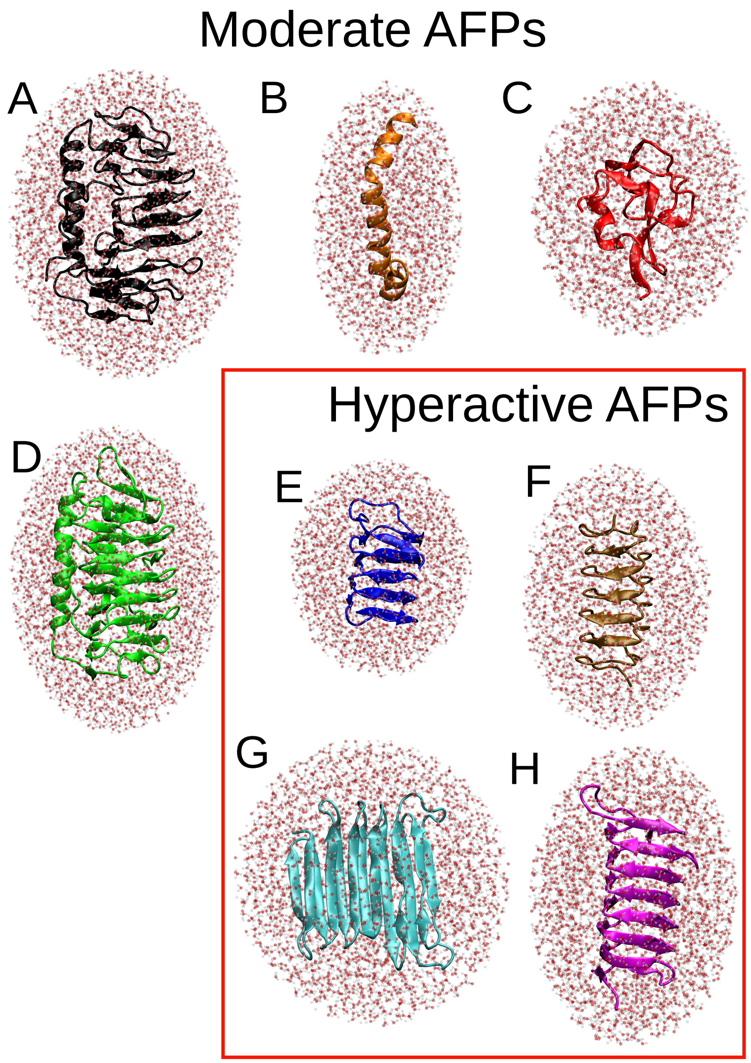

Figure S1: Representative structures of the eight proteins analyzed are reported with a cartoon representation of the backbone and their hydration-shell solvent molecules with a ball and stick representation. The values of the axes used to model the proteins are reported in Table S1, along with the hydration-shell densities. A. *Tis*AFP6, B. *Pa*AFP, C. *Za*AFP, and D. *Tis*AFP8 are moderately-active AFPs with antifreeze activity $<$ 1 K. E. *Cf*AFP337, F. *Tm*AFP, G. *Ri*AFP, and H. *Cf*AFP501 are hyperactive AFPs with antifreeze activity $>$ 1 K.

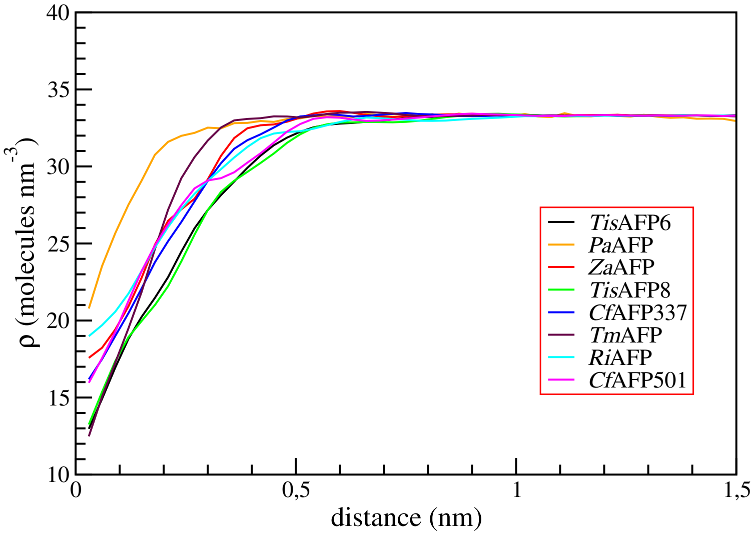

Figure S2: Layer density profile of the solvation SPC molecules as a function of the distance from the protein ellipsoid surface for the eight AFPs.

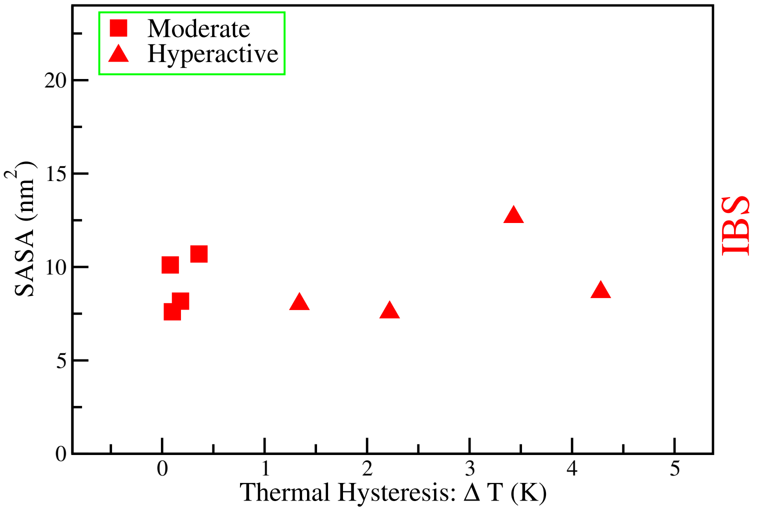

Figure S3: Solvent accessible surface area of the IBS of the eight proteins as a function of the antifreeze activity.

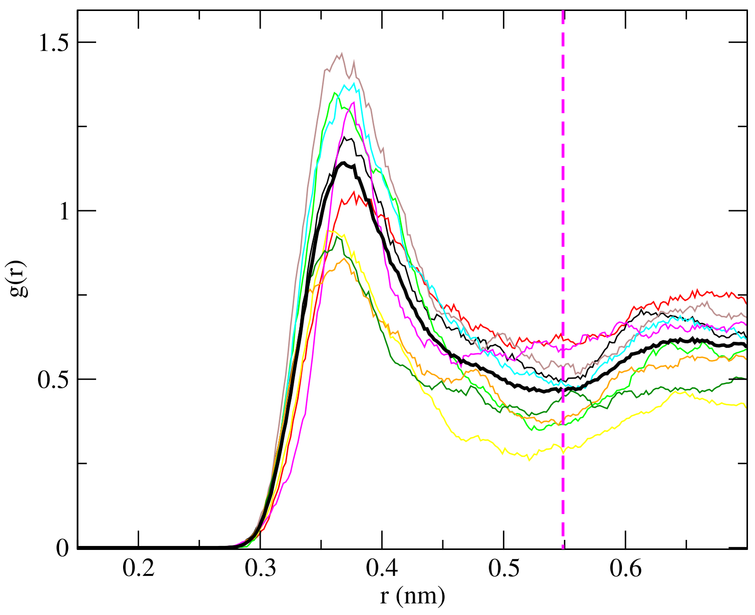

Figure S4: Radial distribution function, g(r), of water molecules around the aliphatic carbon atoms at the surface of the protein. The different g(r) profiles were calculated for several proteins and several surfaces and are represented with different colours. The black thick line is the average over all the g(r) reported in the figure. It can be seen that the local minimum after the first peak is, on average, at around 0.55 nm. Hence, we chose a 0.55 nm cut-off to define the first hydration layer for the calculation of the solvent-density around a given surface, ρ_{surf}, in order to maximize local variations in hydration density between different surfaces.

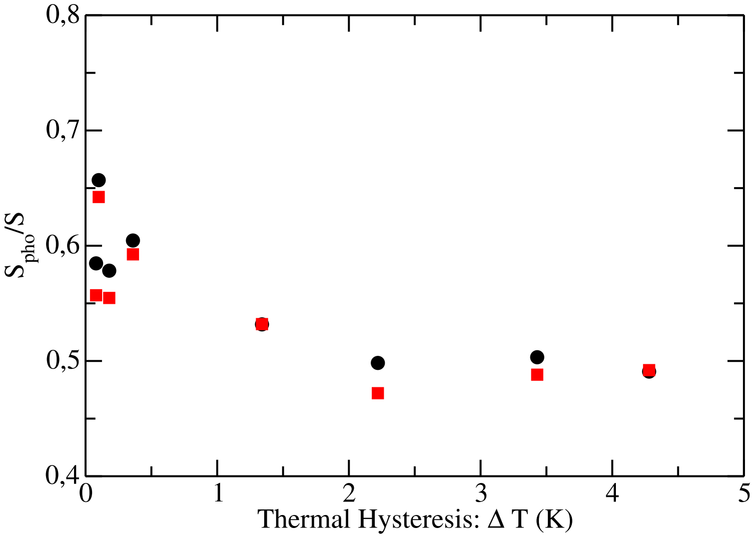

Figure S5: The fraction of hydrophobic SASA is calculated on the crystal structures of all proteins using two different definitions of hydrophobicity: the one based on partial charges (red) and the one based on the chemical nature of the groups to which the atoms belong (black).

D Supplementary Tables

Table S1: Average values of the protein ellipsoid axes (a, b, c) lengths (nm), mean solvent density ρ_{shell} within the accessible volume of the hydration shell (molecules per nm^3) for all studied proteins.

Protein	$\langle a \rangle$	$\langle b \rangle$	$\langle c \rangle$	ρ_{shell}
*Tis*AFP6	1.30	1.68	2.62	37.14
*Pa*AFP	0.48	0.61	3.25	35.04
*Za*AFP	1.06	1.15	1.52	35.70
*Tis*AFP8	1.31	1.67	2.63	36.72
*Cf*AFP337	1.11	1.25	1.60	36.02
*Tm*AFP	0.90	1.01	2.03	36.10
*Ri*AFP	0.86	1.97	2.15	36.29
*Cf*AFP501	1.17	1.27	2.14	36.25

Note: The three axes are averaged over 100 ns (100 000 structures). The mean solvent density ρ_{shell} is calculated according to eqn 1 and 2 of the main text. The error on ρ_{shell} is ≈0.2 % and is evaluated through the standard error of its mean calculated over 3 subtrajectories.

Table S2: Density-increment in the hydration shell relative to the bulk density (η).

Water Model – Force Field	TIP4P/2005 – AMBER 03	SPC – OPLS-AA
η for CfAFP501	0.092	0.088
η for ZaAFP	0.075	0.071

References

(1) Del Galdo, S.; Marracino, P.; D'Abramo, M.; Amadei, A. In silico characterization of protein partial molecular volumes and hydration shells. *Phys. Chem. Chem. Phys.* **2015**, *17*, 31270–31277.

(2) Eisenhaber, F.; Lijnzaad, P.; Argos, P.; Sander, C.; Scharf, M. The double cubic lattice method: efficient approaches to numerical integration of surface area and volume and to dot surface contouring of molecular assemblies. *J. Comput. Chem.* **1995**, *16*, 273–284.

(3) Del Galdo, S.; Amadei, A. The unfolding effects on the protein hydration shell and partial molar volume: a computational study. *Physical Chemistry Chemical Physics* **2016**, *18*, 28175–28182.
